# Investigation of drowning characteristics and its burden in Hormozgan and Bushehr provinces 

**Published:** 2022-12

**Authors:** Sobhan Arayesh, Mohammad Taghi Bastami, Ameneh Marzban

**Affiliations:** ^*a*^ Head of Relief and Rescue of Red Crescent Society Operations, Ilam, Iran.; ^*b*^ Department of Health in Disasters and Emergencies, School of Health Management and Information Sciences, Iran University of Medical Sciences, Tehran, Iran.

## Abstract

**Background:**

Drowning is one of the most important health problems that is often neglected. Mortalities related to this type of incident are the third leading cause of death due to unintentional injuries in the world, after traffic injuries and falls. This study examined the drowning characteristics and its resulting burden, as one of the important indicators in prioritizations.

**Methods:**

This study was performed using the data of deaths due to drowning in residents of Hormozgan and Bushehr provinces which were obtained from the death registration system. First, with the help of descriptive statistics, the characteristics of drowning were described and then the burden was calculated using the standard method of the World Health Organization (WHO).

**Results:**

In 2018, of all residents of the two provinces, 254 people (78.69% male and 21.31% female) died of drowning. Their mean age was 27.15 years (standard deviation of 8.67). Most of the drowning cases (79.11%) were reported at sea and in September (41.36%). The death rate due to drowning was estimated to be 4.27 per 100,000 population and the number of years lost was 4521 years (78.34 per 100,000 people), most of which were in the age group of 30 years.

**Conclusions:**

Due to the high prevalence of this incident, especially in the age range of 18-30 years, which constitutes the young population who are the creators of a better future, it is necessary to implement preventive programs and measures in this regard. It is useful to inform society and increase the awareness of dangers of going to the beach by holding meetings and workshops and preparing educational brochures.

**Keywords:**

Drowning, Burden, Hormozgan, Bushehr

